# Pure hydroxyapatite synthesis originating from amorphous calcium carbonate

**DOI:** 10.1038/s41598-021-91064-y

**Published:** 2021-06-02

**Authors:** Michika Sawada, Kandi Sridhar, Yasuharu Kanda, Shinya Yamanaka

**Affiliations:** 1 Department of Applied Sciences, Muroran Institute of Technology, Hokkaido, 050-8585 Japan; 2 Department of Food Science, Fu Jen Catholic University, New Taipei City, 242 05 Taiwan

**Keywords:** Chemistry, Materials science

## Abstract

We report a synthesis strategy for pure hydroxyapatite (HAp) using an amorphous calcium carbonate (ACC) colloid as the starting source. Room-temperature phosphorylation and subsequent calcination produce pure HAp via intermediate amorphous calcium phosphate (ACP). The pre-calcined sample undergoes a competitive transformation from ACC to ACP and crystalline calcium carbonate. The water content, ACC concentration, Ca/P molar ratio, and pH during the phosphorylation reaction play crucial roles in the final phase of the crystalline phosphate compound. Pure HAp is formed after ACP is transformed from ACC at a low concentration (1 wt%) of ACC colloid (1.71 < Ca/P < 1.88), whereas Ca/P = 1.51 leads to pure β-tricalcium phosphate. The ACP phases are precursors for calcium phosphate compounds and may determine the final crystalline phase.

## Introduction

Hydroxyapatite (HAp; Ca_10_(PO_4_)_6_(OH)_2_) is a form of calcium phosphate. It is the main inorganic mineral component of teeth and bones. HAp can be chemically synthesised as a biomaterial such as artificial tooth roots and bone filling agents^1,2^. Furthermore, HAp has been applied as an adsorbent for chromatography^3^, decomposition catalyst^4^, and catalyst in reduction of nitrophenol^5^.

Much effort has been devoted to the chemical synthesis of HAp due to its tremendous biomedical applications. Recently, many studies are highly centred on HAp synthesis by hydrothermal^6^, sol–gel^7^, and chemical precipitation methods^8,9^. Likewise, a recent investigation by Batista*, *et al*.*^10^ obtained HAp with a yield of 89.25% using combustion synthesis at an optimum temperature of 650 °C for 30 min with urea at pH of 2. Another study by Sirait et al*.*^11^ employed the precipitation method using phosphoric acid as a phosphate source and heated at 600 °C for 4 h under alkaline conditions. However, all these methods employed high temperature, pressure, and extreme pH for synthesis of pure HAp^12^.

The standard synthesis of HAp requires soluble calcium salts^9^. The calcium phosphate reaction can occur under mild conditions. The reaction can also be conducted in with different sources of calcium in the solid-state^13,14^. However, a subsequent purification step is necessary to eliminate the associated anions. On the one hand, calcium carbonate is a promising calcium source due to the absence of interfering anions. Moreover, it has poor water solubility (solubility products of calcite is log*K*_SP,C_ =  − 8.48, aragonite log*K*_SP,A_ =  − 8.34, and vaterite log*K*_SP,V_ =  − 7.91)^15^. Besides this, the use of calcium carbonate as a starting source further transforms unreacted calcium carbonate into calcium oxide prior to HAp formation^16,17^.

To improve the solubility of calcium carbonate, Qi et al*.*^18^ reported HAp synthesis with different phosphorus sources. Likewise, Minh et al.^19^ successfully precipitated calcium phosphate without the formation of calcium oxide. Unfortunately, the crystal phase of calcium phosphate included not only HAp, but also mono-calcium phosphate monohydrate (Ca(H_2_PO_4_)_2_·H_2_O) and dicalcium phosphate dihydrate (CaHPO_4_·2H_2_O)^19^.

The development of a simple and scalable process to prepare pure HAp under mild reaction conditions has been a challenge. The “biomimetics” approach is one solution. HAp precipitation has been carried out under the coexistence of a soft template such as casein^20^ and chitosan/polyacrylic acid nanogel^21^. Sheikh et al.^22^ demonstrated a biomimetic matrix-mediated synthesis of nano-HAp at room temperature using bovine serum albumin, collagen, or polyvinyl alcohol to control the nucleation and growth of HAp at the nano level.

The concept of non-classical crystallisation via amorphous intermediates has been proposed. One of the most representative calcium compounds are calcium carbonate and calcium phosphate. Crystalline calcium carbonate polymorphs of calcite, aragonite, and vaterite are crystallised via dissolution and re-crystallisation of unstable amorphous calcium carbonate (ACC)^23–26^. Some reports in the field of non-classical crystallisation have described amorphous nano-particles as precursors of crystalline calcium carbonate^27–29^ and calcium phosphate^30,31^. Lotsari*, *et al*.*^32^ showed the evidence on the amorphous calcium phosphate (ACP) phase in vitro and in vivo. At a neutral pH and moderate supersaturation, ACP is often the first-formed deposit, but eventually transforms into the thermodynamically more stable HAp^33,34^. The role of such nanometre-sized ACP as building blocks for calcium phosphate crystals has been debated for many years.

Based on this concept, we propose a new HAp synthesis strategy originating from ACC colloids. We demonstrate that pure HAp or β-tricalcium phosphate (β-TCP; Ca_3_(PO_4_)_2_) is synthesised by a reaction of ACC colloids with an orthophosphoric acid solution at room temperature and a subsequent calcination process. We experimentally confirm that the water content or ACC concentration controls the phase purity of the calcium phosphate compounds. Additionally, we clarify that a competitive phase transformation occurs from ACC to ACP and a crystalline phase of calcium carbonate. We also show that the molar ratio of Ca and P plays a crucial role on the formation of the calcium phosphate phase. Finally, we confirm that powdered ACC, vaterite, or calcite with similar specific surface areas do not provide pure HAp. Only the ACC colloid can be used as the starting source for pure HAp. Thus, HAp may originate from the ACC colloid.

## Experimental

### Materials

Calcium hydroxide (purity > 96.0%), ethanol (> 99.5%), glycerin (> 99.0%), phosphoric acid (> 85.0%), and acetone (> 99.5%) were purchased from Kanto Chemical (Tokyo, Japan) and used as received.

### ACC colloid synthesis

The preparation method for the ACC colloid dispersions is described elsewhere^35^. Briefly, 25.0 g calcium hydroxide was added to 475 g solvent mixture of ethanol and glycerin (7:3 by weight) while stirring at 400 rpm in a reaction vessel. A mixed gas of nitrogen (70 vol%) and carbon dioxide (30 vol%) flowed at a rate of 1 L/min to start the carbonation reaction. The temperature was adjusted to 20 °C during the carbonation reaction. The carbonation reaction was completed when the apparent pH did not change (pH ~ 8.9). Then the unreacted coarse particles were removed by centrifugation at 3,540 g for 20 min to give an ACC colloid dispersion.

The ACC colloid concentration was determined by thermogravimetric analysis (TG/DTA6200N, SII, Chiba, Japan). N_2_ gas was introduced at a rate 80 ml/min, the rate of temperature increase was 10 °C/min, and the concentration was determined by the weight decrease within the temperature range of 20–500 °C. The ACC colloid was diluted to 5 wt% or 1 wt% using the solvent mixture of ethanol and glycerin (7:3 by weight). Then it was subjected to the phosphoric acid treatment described in “Phosphorylation” section.

ACC, vaterite, and calcite powders were prepared from the ACC colloid. The methodology and powder characteristics are described in the supporting information.

### Phosphorylation

The ACC colloid (30 g of 5 wt%) and a 5 wt% phosphoric acid solution were placed in a container. The molar ratio of Ca and P was set to Ca/P = 1.68 (stoichiometric HAp is 1.67). Distilled water or acetone was used as the solvent for the phosphoric acid solution. After stirring at room temperature at 800 rpm for 180 min, the sample was centrifuged at 3540 *g* for 15 min, washed with ethanol, and vacuum dried for 24 h. To identify the crystal phase of the synthetic sample, atmospheric calcination was conducted at 800 °C for 2 h using an electric furnace (FO-200, Yamato Science, Tokyo, Japan).

The 1 wt% ACC colloid was reacted with a 5 wt% phosphoric acid aqueous solution. Subsequent operations were the same as those for the 5 wt% ACC colloid treatment. Notably, the Ca/P ratios were set to 1.51, 1.58, 1.67, 1.71, 1.77, and 1.88.

### Characterisation

The particle morphologies were observed using a scanning electron microscope (SEM, JSM-6380A, JEOL, Tokyo, Japan). The crystal structure was analysed using an X-ray diffractometer (XRD, MultiFlex-120NP, Rigaku, Tokyo, Japan) with Cu Kα radiation (40 kV, 20 mA). The measurement range was 15°–50° at a scan speed of 5°/min. To estimate the surface functional group on calcium compounds, Fourier-transform infrared (FT-IR) spectra (FT/IR-460PlusK, JASCO, Tokyo, Japan) were acquired using a KBr pellet technique with a scan range from 400 to 4000 cm^−1^.

## Results and discussion

### Competitive phase transformation of ACP and crystalline calcium carbonate

**Figure 1:**
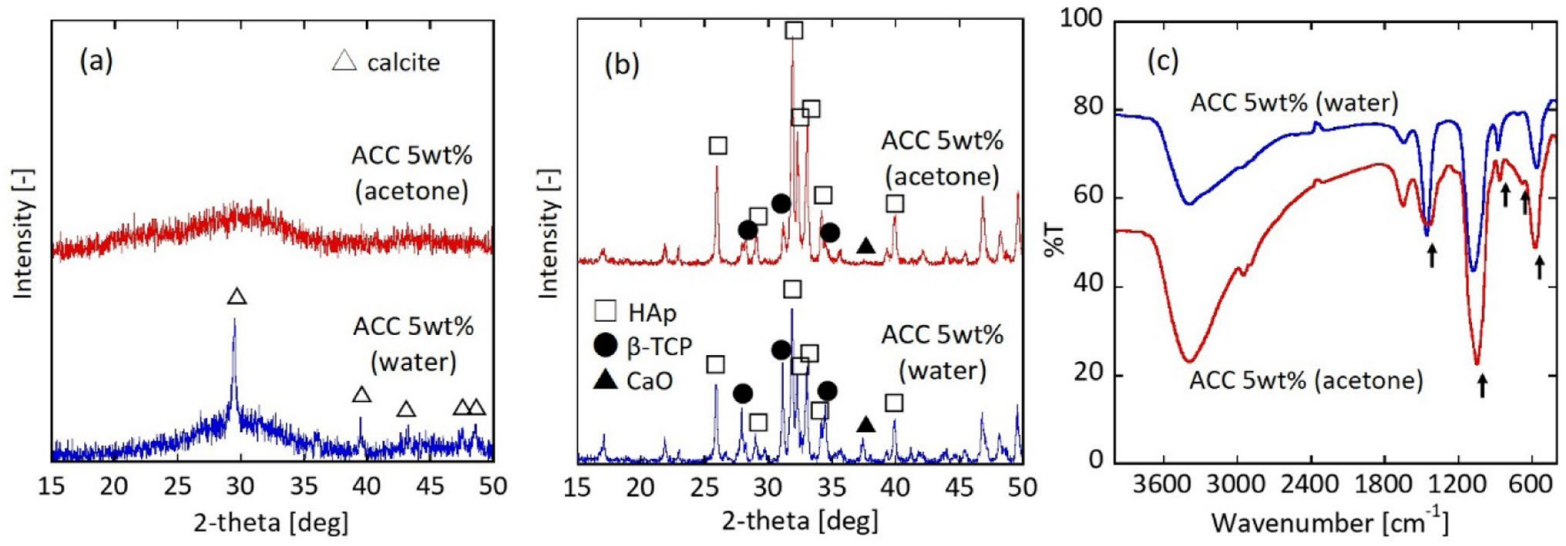
XRD patterns of (**a**) pre-calcined and (**b**) calcined particles. Peak positions in the XRD profiles correspond to those of calcite (JCPDS no. 05-0586), hydroxyapatite (JCPDS no. 09-0432), β-TCP (JCPDS no. 09-0169)), and calcium oxide (JCPDS no. 37-1497). (**c**) FTIR spectra for the pre-calcined sample. Phosphorylation reaction is carried out in water and acetone systems using 5 wt% ACC colloid (Ca/P = 1.68).

We initially examined the phosphorylation reaction in two different systems: water and acetone. Figure 1a shows the XRD patterns of the sample before and after calcination. For the water system (blue line), the pre-calcined sample exhibited a clear calcite peak at 2*θ* = 29.4° and a broad pattern around 30°, indicating that a part of ACC underwent a phase transformation to calcite via dissolution and recrystallisation in water^23–26^ prior to reacting with the phosphate ions. In addition to ACC, the broad pattern suggests the possibility of ACP, which is an intermediate state in crystalline calcium phosphate compounds^31–34^. During the phosphorylation reaction, ACC partially reacted with phosphoric acid to form ACP.

For the water system, a calcination treatment was performed to clarify the crystalline phase of the calcium compounds. The calcined sample contained peaks of HAp, β-TCP, and calcium oxide (Fig. 1b, blue line). The crystalline calcite in the pre-calcined sample was converted to calcium oxide during the calcination treatment. ACP, which was generated when ACC transformed into the crystalline calcium phosphate compounds, occurred simultaneously.

To prevent the ACC transformation into crystalline calcium carbonate, the phosphorylation reaction of the ACC colloid was conducted in an acetone system. The pre-calcined sample was amorphous (ACP or ACC) without a crystal phase (Fig. 1a, red line). Reflecting this XRD result, the main crystalline phase of the calcined sample was HAp (Fig. 1b, red line). However, a slight peak of β-TCP and a minute calcium oxide peak were also observed.

Figure 1c shows the FT-IR spectrum for the pre-calcined sample in the water and acetone systems. The stretching mode at 1000–1100 cm^−1^ and the bending mode at 550–600 cm^−1^ were assigned to the ${\text{PO}}_{4}^{3 - }$ group^36–39^. The doubly degenerate asymmetric stretching mode at 1400–1500 cm^−1^, out of plane bending mode at 840–900 cm^−1^, and doubly degenerate planar bending mode at 650–750 cm^−1^ originated from the ${\text{CO}}_{3}^{2 - }$ groups^40,41^. Regarding the pre-calcined sample, the XRD results suggest the possibility of ACP in addition to the unreacted ACC. During the phosphorylation reaction, ACC partially reacted with phosphoric acid to form ACP. FT-IR spectrum in Fig. 1c denotes that the modes are assigned to the ${\text{CO}}_{3}^{2 - }$ and ${\text{PO}}_{4}^{3 - }$ groups, indicating the pre-calcined sample included ACC and ACP.

**Figure 2:**
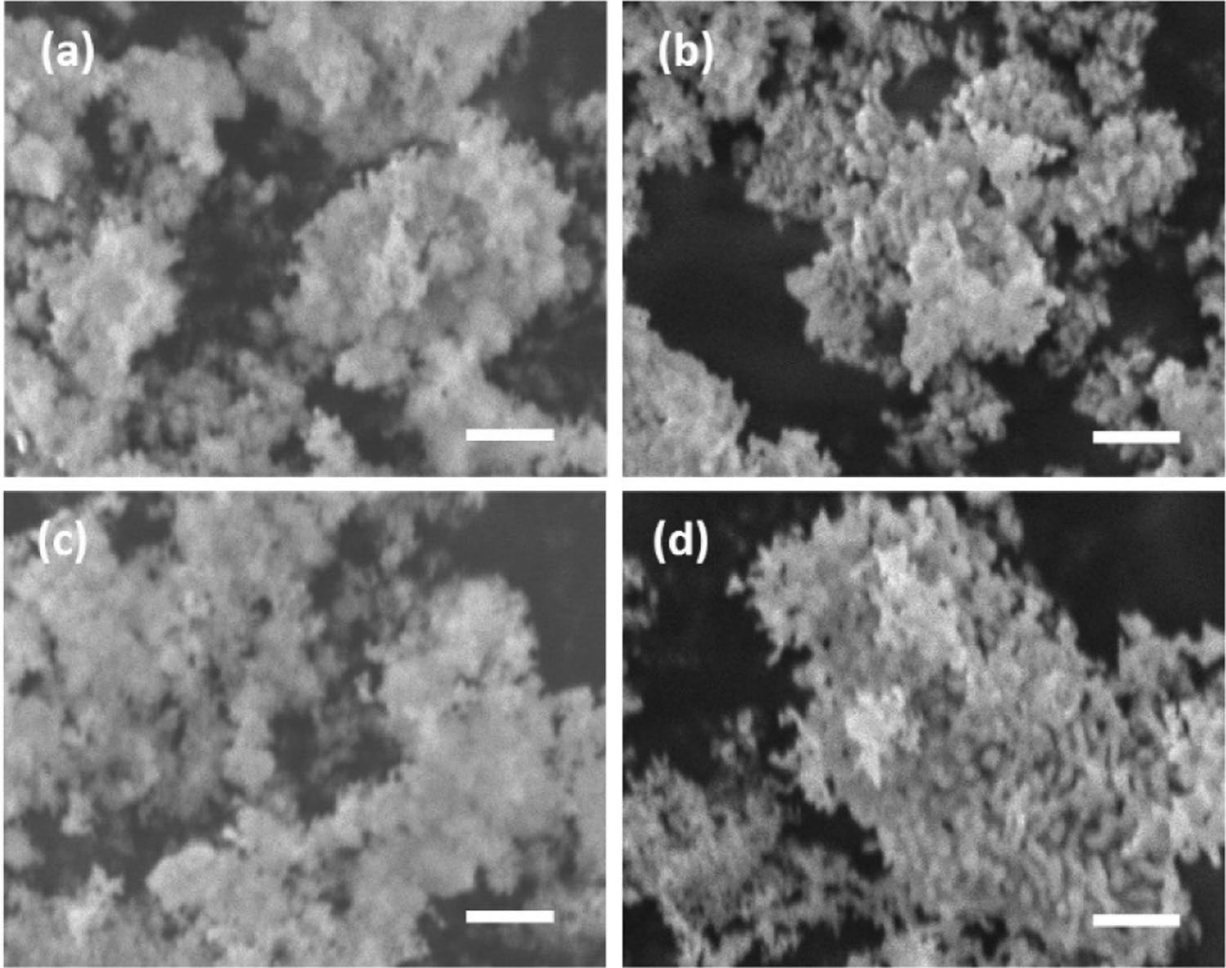
Typical SEM images of the particles phosphorylated in (**a**,**b**) a water and (**c**,**d**) an acetone system. (**a**,**c**) Pre-calcined and (**b**,**d**) calcined samples. Scale bar is 1 μm.

Figure 2 depicts typical SEM images of the sample before and after calcination. The images of the pre-calcine sample (Fig. 2a,c) revealed aggregates of shapeless fine particles. In contrast, the calcined sample had nano-sized particles with clear edges, indicating that ACP was transformed into crystalline calcium phosphate and sustained subsequent particle growth during the calcination treatment.

Since the aqueous system had a higher phase transformation from ACC to calcite, calcium oxide remained as an impurity in the calcined sample. The phase transformation to crystalline polymorphs in the acetone system was inhibited compared with the water system. Thus, the ACC reaction with phosphate ions was prioritised to form ACP. Suppressing the phase transformation of ACC to crystalline calcium carbonate should synthesise pure HAp.

### Pure HAp synthesis

**Figure 3:**
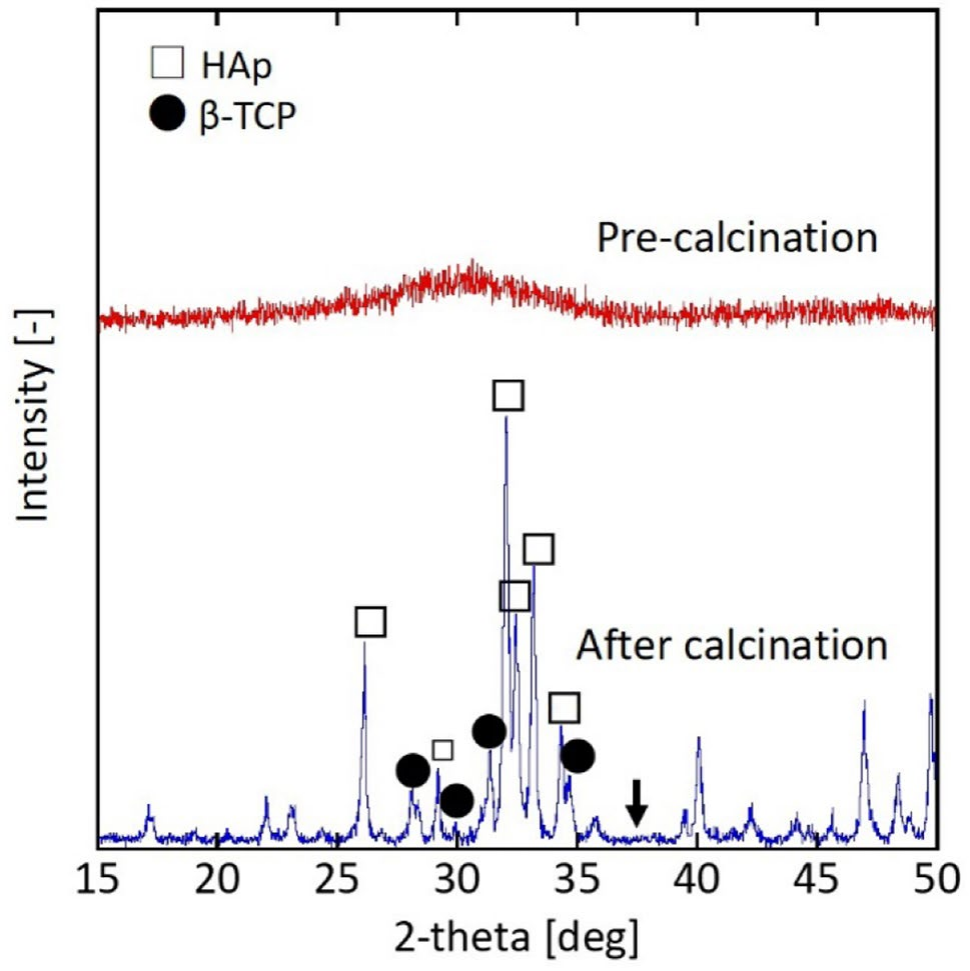
XRD patterns of pre-calcined and calcined particles phosphorylated in the water system using the 1 wt% ACC colloid (Ca/P = 1.67). Arrow denotes the peak position (2*θ* = 37.3°) of calcium oxide.

Here, we assumed the phase transformation rate of ACC (*v*_trs_) depended on the particle concentration (*C* [1/m^3^]), which is expressed from the following Arrhenius-type equation.

1 $$v_{{{\text{trs}}}} = k_{0} {\text{exp}}\left( { - \frac{{E_{a} }}{RT}} \right)C$$

where *E*_*a*_ is the activation energy [J/mol], *k*_0_ is the frequency factor [1/s], *R* is the gas constant [J/(mol·K)], and *T* is the absolute temperature [K]. As described in Fig. 1, the pre-calcined sample contained calcite when the starting calcium source was the 5 wt% ACC colloid, and the phosphorylation reaction occurred in the water system. According to Eq. (1), decreasing the particle concentration should inhibit the phase transformation rate. Therefore, the starting material was the 1 wt% ACC colloid instead of the 5 wt% ACC colloid, and the phosphorylation reaction was performed in the water system. Figure 3 summarises the XRD results before and after calcination.

The crystalline phase was not detected in the pre-calcined sample prepared from the 1 wt% ACC colloid phosphorylated in water. Notably, the calcium oxide peak disappeared in the calcined sample, indicating that the sample was almost ACP. The slight β-TCP peak indicated an insufficient amount of Ca (stoichiometrically Ca/P of β-TCP is 1.50). Consequently, the 1 wt% ACC colloid is a candidate starting source to synthesise a pure HAp phase.

**Figure 4:**
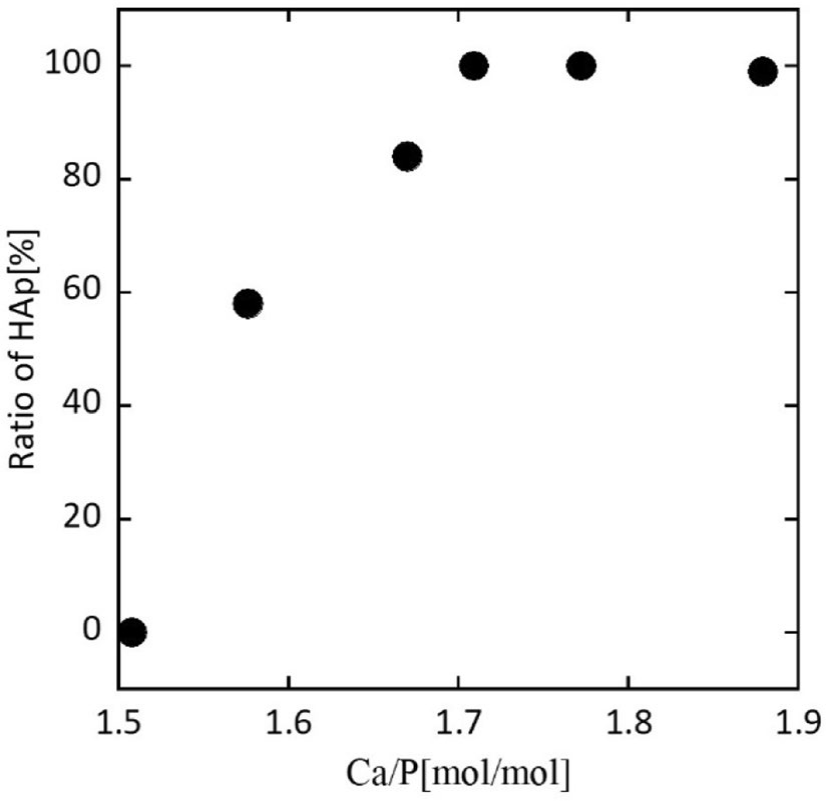
HAp/β-TCP ratio as a function of the Ca/P ratio calculated by the RIR method. Ratio = 0% denotes the crystalline phase is β-TCP. Excess amount of Ca (Ca/P = 1.88) includes 0.6% calcium oxide due to the unreacted ACC.

Next, the molar ratio of Ca/P was adjusted in the phosphorylation reaction (see “ACC colloid synthesis” section). Figure 4 plots the HAp/β-TCP ratio as a function of the Ca/P molar ratio. The HAp ratio was calculated by the Reference Intensity Ratio (RIR) method. The RIR method is based on scaling all diffraction data to the diffraction of standard reference materials^42,43^. For a Ca/P molar ratio of 1.71, the HAp ratio was 1.0, indicating a pure phase of HAp. For Ca/P ratios of 1.51 and 1.58, which were smaller than the stoichiometric ratio for the HAp (Ca/P = 1.67), β-TCP was the coexistence phase. Ca/P = 1.51and Ca/P > 1.71 yielded pure β-TCP and HAp, respectively.

The ACC colloidal particle sizes in the dispersion ranged from 4 to 30 nm^35^. The amorphous and nano-sized ACC is a chemically active substance, which should easily crystallise into a stable phase. These experiments revealed the following points about the phase transformation of ACC during the phosphorylation reaction:

1. Using acetone as a solvent inhibits the dissolution and re-crystallisation of ACC to form the crystalline calcium carbonate.
2. Decreasing the ACC concentration prevents the phase transformation and promotes the ACC reaction with phosphoric acid to form ACP.
3. ACP is transformed to crystalline calcium phosphate compounds, where the main phase of calcium phosphate depends on the Ca/P ratio.

To confirm that the key characteristics to promote the phosphorylation reaction are "nano-particles" and have an "amorphous" structure, phosphorylation was carried out under the conditions of Ca/P = 1.71 and a 1 wt% particle concentration.

### Key factors of calcium sources for the pure HAp synthesis

**Figure 5:**
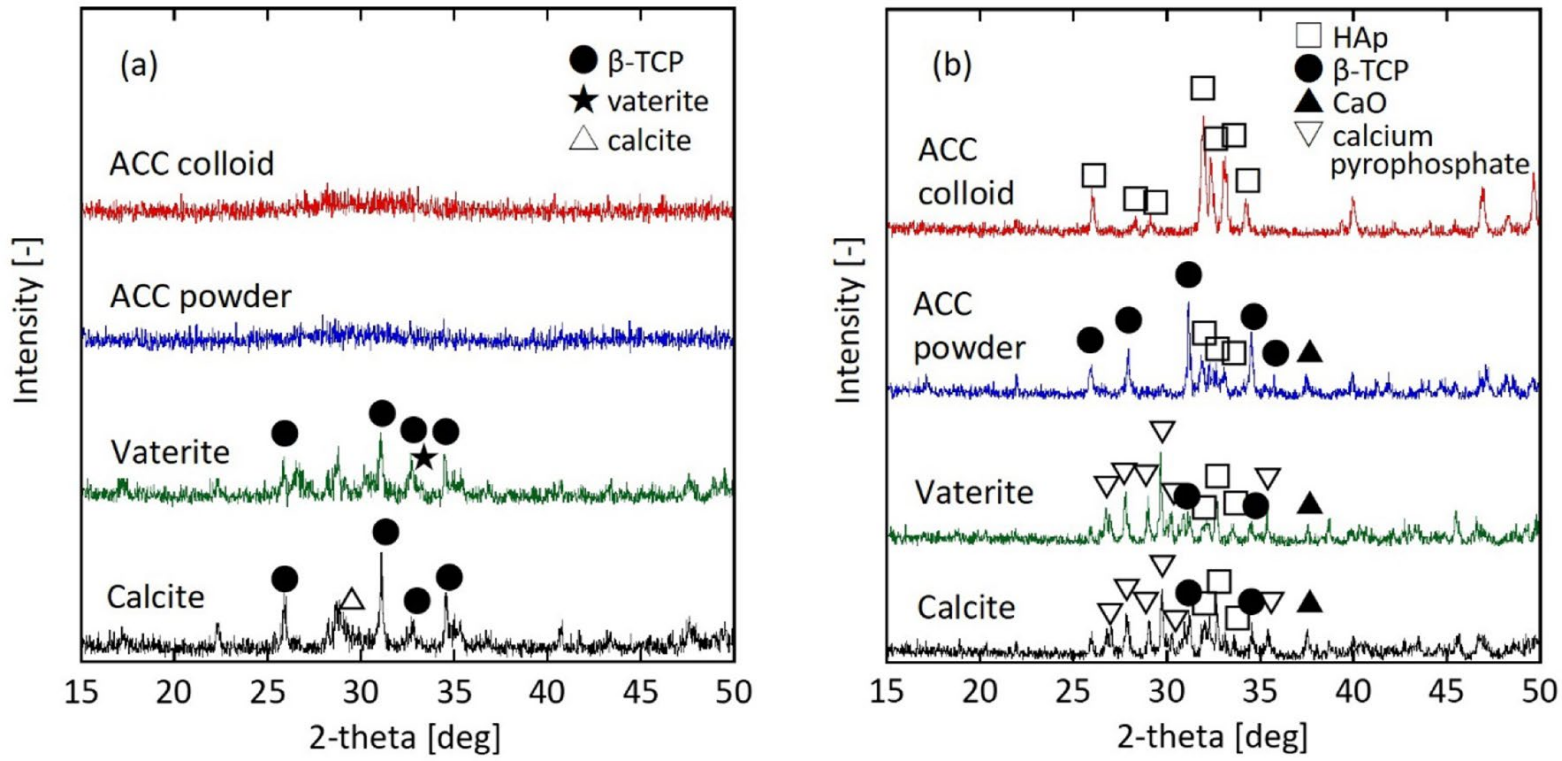
XRD patterns of (**a**) pre-calcined and (**b**) calcined particles. Phosphorylation reaction is performed in a water system using a 1 wt% ACC, vaterite, or calcite powder suspension. Additionally, the reaction with a 1 wt% ACC colloid is also shown. Molar ratio of Ca/P is 1.71.

We used a 1 wt% suspension of ACC, vaterite, or calcite powder (see supporting information) as the starting source in the phosphorylation reaction. Figure 5 shows the XRD powder patterns before and after calcination. A comparison of the ACC colloid with a suspension of ACC powder confirmed that the pre-calcined sample was in the amorphous phase (ACP). For the calcined samples, the 1 wt% ACC colloid produced a pure HAp peak, whereas the 1 wt% suspension of ACC powder mainly gave a β-TCP peak.

According to the Ostwald-Freundlich equation, the solubility is substance specific when the temperature is constant. In contrast, the solubility increases as the particle size decreases^44^ according to the following equation

2 $$\mathrm{ln}\frac{{x}_{a}}{{x}_{a}^{\infty }}=\frac{2\gamma M}{RT\rho r}$$

where *x*_*a*_ [mol/L] is the solubility when the particle radius is *r*, ${x}_{a}^{\infty }$ [mol/L] is the solubility when the particle radius is infinite, γ [N/m] is the surface energy, *M* [g/mol] is the molar mass, *R* [J/(mol·K] is the gas constant, *T* [K] is the absolute temperature, *ρ* [g/cm^3^] is the particle density, and *r* [m] is the particle radius at equilibrium.

The particle size of the ACC colloid was 4–30 nm^35^, whereas that of the ACC powder was 2 μm (see Fig. S1). Because the solubility of ACC 8.4–12.4 times decreases with size, as expressed in Eq. (2), the nano-sized ACC colloid should have a much higher reactivity with phosphoric acid than the micron-sized ACC powder. Additionally, the ACC size is a dominant factor for the final crystalline phase of calcium phosphate. Although this study did not investigate the ACP micro-structure, the results suggested that the final crystalline phase of calcium phosphate compounds depends on the intermediate structure of ACP.

**Figure 6:**
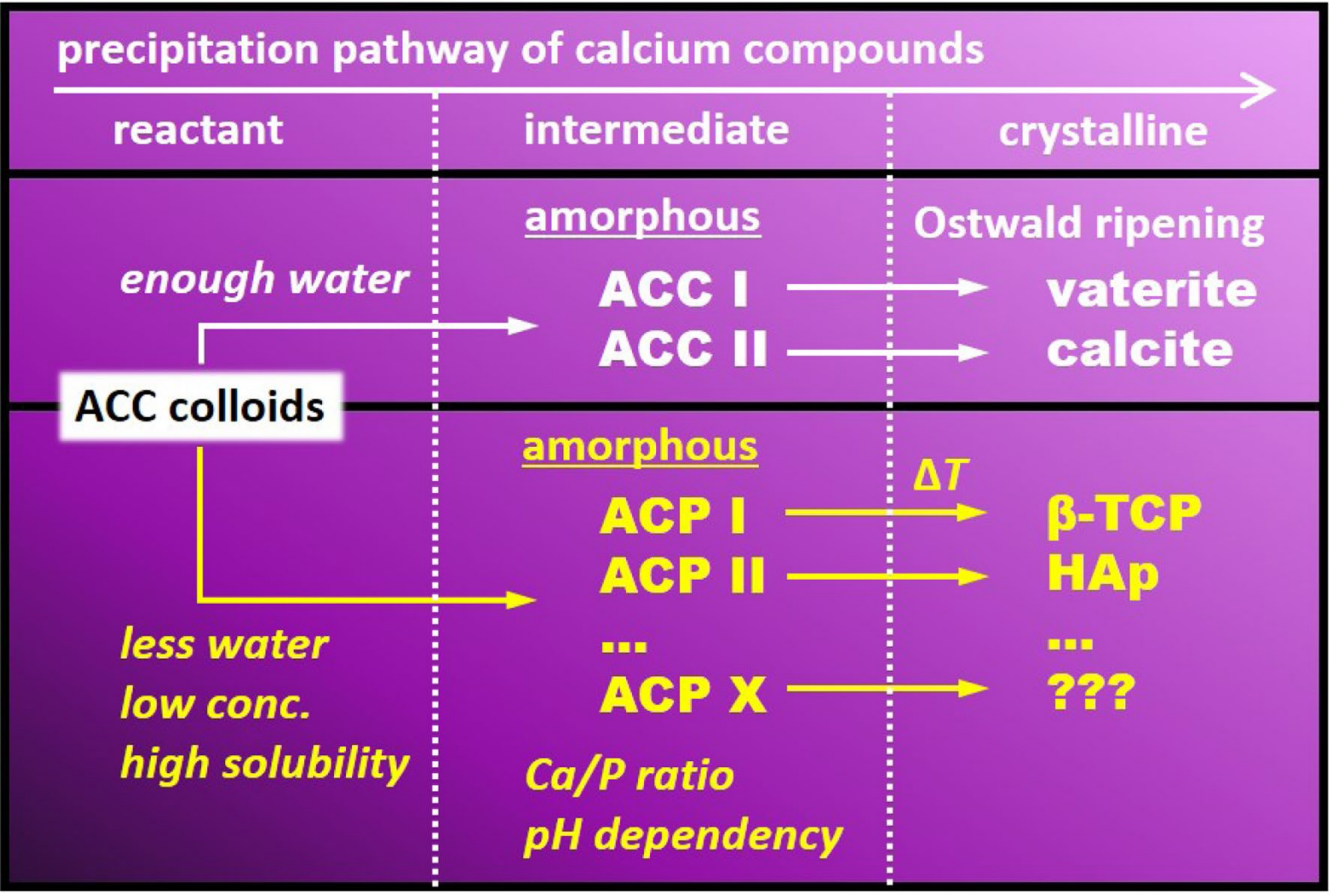
Schematic illustration of calcium phosphate precipitation.

**Figure 7:**
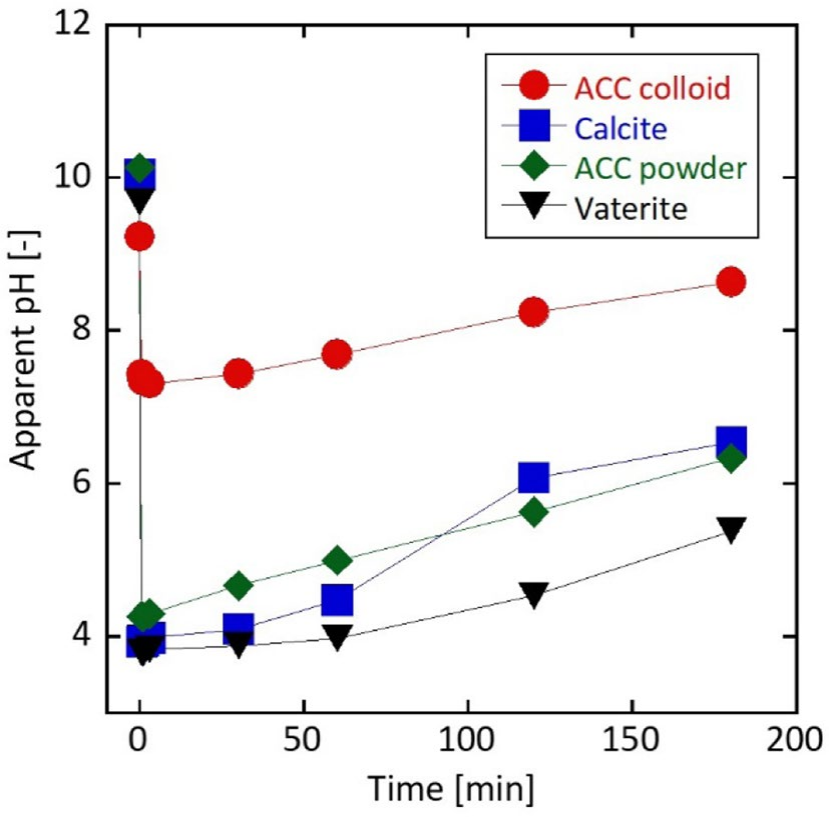
Time dependence of the apparent pH during the phosphorylation reaction conducted in a water system using ACC colloid, powdered ACC, vaterite, and calcite suspensions. Particle concentration is 1 wt%, and the molar ratio of Ca/P is 1.71.

Amorphous nano-particles were a precursor of crystalline calcium carbonate. According to Gebauer et al.^29^, precursor species with different ACC phases give rise to an alternative crystallisation channel. That is, ACC I and II are promising precursors of vaterite and calcite, respectively^29^. Regarding HAp precipitation, Mahamid et al. identified the ACP phase^45^. At a neutral pH and moderate supersaturation, ACP is often the first-formed deposit, but it eventually transforms into the thermodynamically more stable HAp^34^. Thus, at least two types of ACP, which are named ACP I and ACP II, are independently nucleated in the phosphorylation reaction (Fig. 6). ACP I is related to an amorphous phase exhibiting a tricalcium phosphatic ordered structure, while ACP II is related to an HAp ordered structure. Precursor species of different ACP and ACC phases give rise to alternative crystallisation channels. When the system contains sufficient water, the ACC colloid crystallises into calcite and/or vaterite. Notably, the thermodynamically unstable vaterite phase should transform to the stable calcite phase via dissolution and re-crystallisation of vaterite (Ostwald ripening). In contrast, the ACC colloid transforms to the ACP phase in a non-aqueous system (see Fig. 1). At a low ACC colloid concentration, it transforms to the ACP phase (see Fig. 3). Ca/P (see Fig. 5) and reaction pH (see later discussion in Fig. 7) play important roles on the ACP phase. Below the stoichiometric ratio of HAp (Ca/P < 1.67) or at acidic conditions, ACP I may be a main phase. When the Ca/P is above 1.67 or at neutral pH, ACP II may be the main phase. ACP I and ACP II transform to β-TCP and HAp during the calcination process, respectively. Solubility of the reactants is a fundamental property to decide the subsequent route (see later discussion in the last part of text).

Next, we focused on the amorphous part, which is a characteristic of ACC. The phosphorylation reaction was performed using a 1 wt% suspension of crystalline vaterite or calcite powder. The specific surface areas of the synthesised vaterite and calcite were similar to that for the ACC powder (Table S1). The pre-calcined sample contained vaterite or calcite as the starting source (Fig. 5). The calcined sample contained calcium pyrophosphate in addition to the peaks for HAp, β-TCP, and calcium oxide.

Calcium pyrophosphate was generated by dehydration of phosphoric acid. For instance, calcination of calcium hydrogen phosphate dehydrate or calcium hydrogen phosphate (anhydrous salt) in air generated calcium pyrophosphate. The transition from γ- to β-type, and β- to α-type occurred at 700–750 °C and 1140–1150 °C, respectively^46,47^.

From this point of view, the calcination process produced calcium pyrophosphate. Therefore, to predict the reaction of calcium carbonate with phosphoric acid, the apparent pH was measured at 0.5, 1, 3, 30, 60, 120, and 180 min. 0 min denotes the moment when the phosphoric acid aqueous solution was added. The apparent pH at 0 min of each solution was basic (pH = 9 ~ 10), but immediately dropped to become acidic (pH ~ 4) upon the addition of the phosphoric acid, except for the ACC colloid (Fig. 7). When the starting source was the ACC colloid, the pH was clearly higher than those of the other sources, and the pH did not recover to pH ~ 8.5 until 180 min. However, suspensions of the powdered ACC, vaterite, and calcite remained acidic after 180 min of the phosphorylation reaction.

HAp is stable under basic conditions, whereas calcium hydrogen phosphate and calcium hydrogen phosphate dehydrate, which are sources of calcium pyrophosphate, are stable under acidic conditions^48,49^. Thus, calcium pyrophosphate was produced in the calcined sample obtained from a vaterite or calcite suspension.

The solubility product (*K*_SP_) of calcium carbonate (*K*_SP,V_ for vaterite and *K*_SP,C_ for calcite) depends on the absolute temperature (*T*) [K] according to the following relationships^50^. In addition, the temperature-dependency of *K* for ACC (*K*_SP,ACC_) is also shown^51^.

$$\begin{aligned} {\text{vaterite}}:\log K_{{{\text{SP}},{\text{ V}}}} & = - 172.1295 - 0.077993T + \frac{3074.688}{T} + 71.595\log T \\ {\text{calcite}}:\log K_{{{\text{SP}},{\text{C}}}} & = - 171.9065 - 0.077993T + \frac{2839.319}{T} + 71.595\log T \\ {\text{ACC}}:{\text{log}}K_{{{\text{SP}},{\text{ACC}}}} & = \frac{1247.0}{T} - 10.224{ }\left( {289 \leqq T \leqq 343} \right) \\ \end{aligned}$$

At 25 °C, the calculated log*K*_SP,V_ and log*K*_SP,C_ were – 7.91, and – 8.48, respectively, while log*K*_SP,ACC_ was – 6.04. Y. Kojima et al. reported that ACC (CaCO_3_·1.5H_2_O) rapidly dissolves in water (20 °C), and its solubility is 10 times higher than that of calcite after 1 min infiltration^52^. This corresponds to a lower Gibbs energy for dissolution in ACC (38 kJ/mol) compared with calcite (48 kJ/mol)^53^. HAp was less soluble than other calcium phosphate compounds and had a smaller solubility product of log*K*_SP, HAp_ (− 56.9 to − 52.8 at 20 °C^54^, and − 53.51 to − 53.41 at 25 °C^55^).

The dissolution of calcium carbonate is a key point to precipitate HAp. The ACC colloid is an excellent source for ACP precipitation, and subsequent calcination produces pure HAp or the β-TCP phase.

## Conclusion

This study used the ACC colloid as a starting source for calcium phosphate compounds. The phosphorylation reaction performed in the aqueous system partially confirmed the phase transformation from ACC to calcite. This transformation was inhibited by the phosphorylation reaction in an acetone system or using a low concentration of the ACC colloid. After calcination treatment, the main phase of the reaction was HAp. When a 1 wt% ACC colloid was used as the starting source and the molar ratio of Ca/P was adjusted to > 1.71 or 1.51, pure HAp or β-TCP was respectively synthesised. The precursor species of the different ACP phases gave rise to alternative crystallisation channels: ACP I and II. ACP I and II may be a precursor for β-TCP and HAp, respectively.

To investigate the characteristics of the ACC colloid, we conducted a phosphorylation reaction experiment using powdered ACC, vaterite, or calcite suspensions. The following findings were obtained.

1. Only the ACC colloid synthesised pure HAp, indicating that nano-sized particles are important due to their higher solubility product.
2. Phosphorylation with calcite or vaterite did not synthesise pure HAp because the solubilities of calcite and vaterite are inferior to that of ACC.

## Supplementary information

Supplementary Information.
