# Supplementary material for: Pure hydroxyapatite synthesis originating from amorphous calcium carbonate
Source: Sci Rep. 2021 Jun 2;11:11546. doi: 10.1038/s41598-021-91064-y (PMC8173018; doi:10.1038/s41598-021-91064-y)
Supplement: Supplementary file 1 — Supplementary Information. [file 41598_2021_91064_MOESM1_ESM.pdf]

**Electronic Supplementary Information**

# Pure hydroxyapatite synthesis originating from amorphous calcium carbonate

Michika Sawada [a], Kandi Sridhar [b], Yasuharu Kanda [a], Shinya Yamanaka [a]*

[a] Department of Applied Sciences, Muroran Institute of Technology, 050-8585 Hokkaido, Japan

[b] Department of Food Science, Fu Jen Catholic University, New Taipei City – 242 05, Taipei, Taiwan

Corresponding author. Tel.: +81 143 46 5747; fax: +81 143 46 5701

E-mail address: syama@mmm.muroran-it.ac.jp (S. Yamanaka)

***Experimental procedure for ACC, vaterite, and calcite powder***

ACC powder: The synthesised ACC colloid was diluted with ethanol and stirred at 400 rpm for 4 days at 20 °C. After removing the supernatant by centrifugation at 3,540 *g* for 20 min, a transparent gel remained in the bottom of the centrifuge tube. The obtained gel was washed with ethanol and centrifuged under the same conditions. This treatment was repeated twice. After vacuum drying for 24 h, the obtained coarse translucent particles were ground using a planetary ball mill (P-7, Fritsch, Germany). Translucent particles (2.0 g) and 12.0 g of 3.0 mm yttria-stabilised zirconia balls (Nikkato, Osaka, Japan) were put into a stainless-steel pot with a volume of 45 $cm^3$ and a diameter of 39.95 mm. Atmospheric grinding was carried out at a revolution speed of 400 rpm. A 20 min grinding and 10 min rest cycle to prevent an excessive increase in temperature was repeated thrice for a total grinding time of 60 min. Then the ACC powder was directly collected.

Vaterite powder: Calcium hydroxide (25.0 g) was added to 475 g solvent mixture of ethanol and glycerin (77:23 by weight). The other treatments to obtain the ACC colloid were the same as those described in the main text. Next, the ACC colloid was diluted with ethanol and stirred [Ref. S1] at 400 rpm for 6 days at 20 °C. The colloid became a clouded solution. The supernatant was subsequently removed by centrifugation at 3,540 *g* for 20 min. Washing the sediment with ethanol twice and vacuum drying for 24 hours gave vaterite powder.

Calcite powder: The ACC colloid was left for 1 month in an incubator at 20 °C. The other treatments were the same as those for the vaterite powder.

For the phosphorylation reaction, ACC, vaterite, or calcite powder was suspended in a mixed solvent of ethanol-glycerin (7:3 by weight). The concentration was set to 1 wt%.

***Characterisation***

The specific surface area was measured by nitrogen gas adsorption based on the multi-point BET method. The analysis was conducted on Autosorb-1-c/MK2 (Qantachrome, USA). Prior to the measurements, the samples were degassed for 2 h at 300 °C under a vacuum to remove the adsorbed solvent molecules. The particle morphologies were observed using a scanning electron microscope, SEM (JSM-6380A, JEOL, Tokyo, Japan). The crystal structure was analysed using an X-ray diffractometer, XRD (MultiFlex-120NP, Rigaku, Tokyo, Japan). The measurement range was 15–50° at a scan speed of 5°/min.

***Characteristics of ACC, vaterite, and calcite powder***

Figure S1 shows the SEM images of the ACC, vaterite, and calcite powder. Their particle sizes were several hundred nm to sub-micron. The average particle size based on the number obtained from 250 particles from SEM observations of the ACC powder was 2 µm. The specific surface areas of ACC, vaterite, and calcite powder were 123, 135, and 163 $m^2/g$, respectively (Table S1). The XRD patterns in Fig. S2 revealed a broad peak in the ACC powder. The peak positions in the XRD profiles corresponded to those of the calcite (JCPDS no. 05-0586) and vaterite (JCPDS no. 33-0268) phase for the crystalline powder.

**Reference**

[S1] S. Yamanaka, Y. Sugawara, T. Oiso, T. Fujimoto, Y. Ohira, Y. Kuga, CrystEngComm, 17, 1773 (2015).

**Table S1** Specific surface area of the synthesised powders

| Polymorphs | Specific surface area [$m^2/g$] |
|---|---|
| ACC | 123 |
| Vaterite | 135 |
| Calcite | 163 |

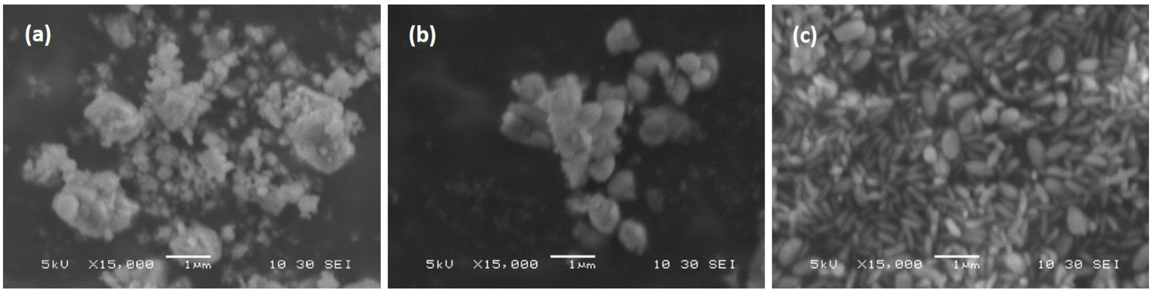


**Figure S1**. Typical SEM images of (a) ACC, (b) vaterite, and (c) calcite powder.

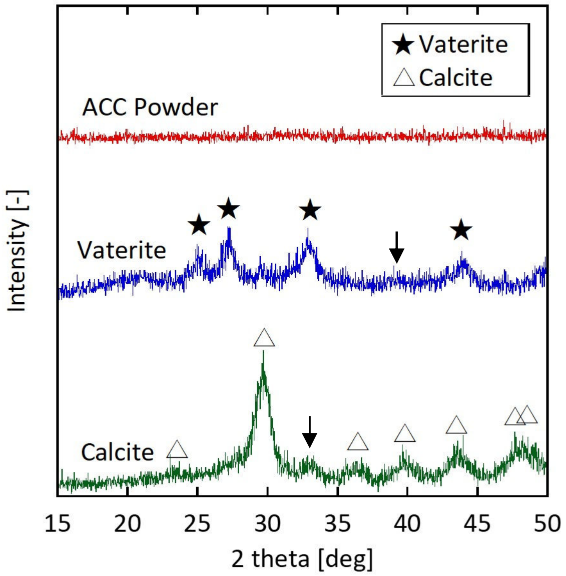


**Figure S2**. XRD patterns of ACC, vaterite, and calcite powder. For calcite, arrow denotes that there is a minute amount of vaterite powder, and vice versa for vaterite.
